# Burden of Infections in Early Life and Risk of Infections and Systemic Antibiotics Use in Childhood

**DOI:** 10.1001/jamanetworkopen.2024.53284

**Published:** 2025-01-06

**Authors:** Nicklas Brustad, Frederik Buchvald, Signe Kjeldgaard Jensen, Julie Nyholm Kyvsgaard, Nilo Vahman, Jonathan Thorsen, Ann-Marie Malby Schoos, Ulrikka Nygaard, Nadja Vissing, Jakob Stokholm, Klaus Bønnelykke, Bo Chawes

**Affiliations:** 1 Copenhagen Prospective Studies on Asthma in Childhood, Herlev and Gentofte Hospital, University of Copenhagen, Copenhagen, Denmark; 2 Department of Pediatrics, Rigshospitalet, Copenhagen, Denmark; 3 Department of Pediatrics, Slagelse Hospital, Slagelse, Denmark; 4 Department of Food Science, University of Copenhagen, Frederiksberg, Copenhagen, Denmark; 5 Department of Clinical Medicine, Faculty of Health and Medical Sciences, University of Copenhagen, Copenhagen, Denmark

## Abstract

**Importance:**

A high infection burden in early childhood is common and a risk factor for later disease development. However, longitudinal birth cohort studies investigating early-life infection burden and later risk of infection and antibiotic episodes are lacking.

**Objective:**

To investigate whether early-life infection burden is associated with a later risk of infection and systemic antibiotic treatment episodes in childhood.

**Design, Setting, and Participants:**

This longitudinal cohort study of children from birth to age 10 or 13 years included data from the Danish population-based Copenhagen Prospective Studies on Asthma in Childhood (COPSAC) birth cohort between November 2008 to November 2010. Children were monitored for infection diagnoses and systemic antibiotic prescriptions from national databases until February 1, 2024, by which time they had completed the age 10- or 13-year visit. Children with immune deficiencies or congenital diseases were excluded.

**Exposures:**

Daily diary-registered common infection episodes of cold, acute otitis media, tonsillitis, pneumonia, gastroenteritis, and fever episodes from birth to 3 years.

**Main Outcome and Measures:**

After age 3 years, the incidence of moderate to severe infection diagnoses and systemic antibiotic prescriptions were estimated using adjusted incidence rate ratios (AIRRs) calculated from quasi-Poisson regression models. All analyses were adjusted for social and environmental confounders.

**Results:**

A total of 614 children (317 male [51.6%]) with diary data from birth to 3 years had completed follow-up until age 10 or 13 years. No differences in baseline characteristics between the children having vs not having available diary data were noted. Children with a high vs low burden of diary-registered infections between birth and 3 years (ie, equal to and above vs below the median of 16) had an increased risk of later moderate to severe infections (181 vs 87 episodes; AIRR, 2.39; 95% CI, 1.52-3.89) and systemic antibiotic treatments (799 vs 623 episodes; AIRR, 1.34; 95% CI, 1.07-1.68) until age 10 or 13 years. Each diary infection episode also increased the later risk of moderate to severe infections (AIRR, 1.05; 95% CI, 1.02-1.08) and systemic antibiotic treatments (AIRR, 1.02; 95% CI, 1.01-1.04). Subtype analyses showed significant associations between each cold, acute otitis media, pneumonia, gastroenteritis, and fever episode between birth and 3 years and risk of later moderate to severe infections or systemic antibiotic treatments.

**Conclusions and relevance:**

This longitudinal cohort study suggests that early-life infection burden may continue throughout childhood and is associated with later antibiotic treatments independent of social and environmental risk factors. These findings are important for prognosis and follow-up of children experiencing a high burden of common infections in early life.

## Key Points

**Question:**

Does early-life infection burden track throughout childhood and into adolescence?

**Findings:**

In this cohort study of 614 children with daily diary information on common infection load in early life, the overall infection burden in early life was associated with increased risks of moderate to severe infections and antibiotic treatments later in childhood.

**Meaning:**

These findings are important for prognosis and follow-up of children experiencing a high burden of common infections in early life.

## Teaser

This cohort study examines whether early-life infection burden is associated with a later risk of infection and systemic antibiotic treatment episodes in childhood.

## Introduction

Infection burden in early childhood is associated with substantial health-related costs^1^ and an increased morbidity and mortality rate worldwide.^2,3^ The number of infections acquired during childhood has been shown to increase the risk of later development of atopic diseases,^4,5^ cardiometabolic risk,^6^ and mental disorders,^7^ emphasizing the importance of identifying and closely monitoring children experiencing frequent infections throughout childhood. For an optimized understanding of infection susceptibility in otherwise healthy children, it is important to consider risk factors such as environmental and social circumstances, which have been implicated in the recurrence of respiratory infections that constitute the majority of infections in childhood.^8,9,10^ To investigate whether the burden of infections in early life persist throughout childhood, comprehensive longitudinal studies are needed alongside detailed information on relevant exposures. We conducted such a study within the Copenhagen Prospective Studies on Asthma in Childhood 2010 (COPSAC2010) cohort in which children were followed from birth to age 10 or 13 years.

Here, we combine unique data on common infections during the first 3 years of life with subsequent longitudinal tracking of physician-diagnosed infections and systemic antibacterial treatments among the children with the aim of examining the association between burden of early and late childhood infections. Our hope is that the findings provide pediatricians with important clinical knowledge about the trajectories of infection burden during childhood that are useful for prognosis and treatment of children who encounter a high burden of infections in early life.

## Methods

### Study Population

The characteristics of the study population and the enrollment procedure of the population-based COPSAC2010 clinical, single-center, mother-child cohort have previously been described in detail.^11^ Briefly, 736 healthy women were recruited at gestational week 24 between November 2008 and November 2010 and followed up longitudinally in the COPSAC clinic together with their children. This cohort study was approved by the Danish Ethics Committee and the Danish Data Protection Agency. Both parents gave written informed consent before enrollment. We followed the Strengthening the Reporting of Observational Studies in Epidemiology (STROBE) reporting guideline for cohort studies.

### Diary-Registered Infection Burden in Children From Birth to 3 Years

During the age period of birth to 3 years, detailed daily symptom and medication diaries were completed by the parents for each child, supported by both scheduled and acute care visits to the COPSAC clinic. The diary registrations included symptoms of cold (indicative of upper respiratory tract infection symptoms); gastroenteritis (manifested as acute diarrhea and/or vomiting); any fever (>38 °C); and physician-diagnosed acute otitis media, acute tonsillitis, and pneumonia (reflecting lower respiratory tract infections [LRTIs]).^12^ Study physicians reviewed the registrations at each scheduled visit to the research clinic at age 1 week, 1 month, 3 months, 6 months, 1 year, 18 months, 2 years, 30 months, and 3 years in addition to acute care visits to validate symptoms and diagnoses, ensuring accuracy and reliability. All episodes were entered into the COPSAC database and double-checked.

### Moderate to Severe Infections and Systemic Antibiotic Treatments From Age 3 to 10 or 13 Years

From age 3 to 10 or 13 years, the children were followed up at scheduled clinical visits, including longitudinal monitoring of prescriptions and diagnoses obtained via parent interviews and medical record checks until February 1, 2024, when the children had undergone either the age 10- or 13-year planned clinical visits. Hence, all medication prescriptions and infection diagnoses were manually entered into the COPSAC database for every child until follow-up. The following diagnoses representing a moderate to severe infection episode were extracted from the database: sepsis, pneumonia, acute gastroenteritis, pyelonephritis, meningitis or encephalitis, cellulitis, soft-tissue infection or herpes zoster, septic arthritis, osteomyelitis, and endocarditis (eTable 1 in Supplement 1 provides specific *International Statistical Classification of Diseases, Tenth Revision* [*ICD-10*] codes). The list was inspired by a previous large cohort study investigating the risk of serious infections among infants.^13^ Furthermore, we extracted all antibacterial medications for systemic use administered to the children (Anatomical Therapeutic Chemical codes J01.X).

### Acute Airway Virus Samples From Birth to Age 3 Years

For children with 3 diary-registered consecutive days of troublesome lung symptoms defined as cough, wheeze, or dyspnea during the first 3 years of life, the COPSAC clinic obtained nasopharyngeal aspirate samples for viral identification with polymerase chain reaction testing as previously described.^14^ The viral airway pathogens rhinoviruses, respiratory syncytial virus (RSV), and enteroviruses were analyzed.

### Covariates

Available covariates for the cohort that potentially could influence risk of infections were sex^15^; delivery mode^10^; maternal smoking during pregnancy^16^; Apgar score at 1 minute^17^; child hospitalization at birth^18^; embedded fish oil and vitamin D interventions in pregnancy (2 × 2 factorial design of 2.4 g/d n-3 long-chain polyunsaturated fatty acids vs placebo^19^ and 2800 vs 400 IU/d of vitamin D^20,21^); social circumstances, including maternal age, education, and income^10^; time to start of day care^10^; number of older siblings^10^; exposure to pets with fur during the first year of life^22^; and living environment (urban vs rural).^23^ These covariates were included in our analyses as potential confounders.

### Statistical Analysis

All common infections from the daily diaries between birth and 3 years were combined into a single variable, reflecting the total number of infection episodes lasting at least 3 days with a minimum of 3 days between each episode. Children were excluded from the analysis if they had any congenital disease (eg, primary ciliary dyskinesia) or diagnosed immunodeficiencies that could affect infection susceptibility. For later moderate to severe infection diagnosis and systemic antibiotic treatment variables until age 10 or 13 years, all infections and systemic antibiotics occurring before the age of 3 years were excluded. Furthermore, we created a specific pneumonia variable based on *ICD-10* codes J12.xxx to J18.xxxx from age 3 to 10 or 13 years. Finally, we created a variable summarizing the total number of each virus type from the acute nasopharyngeal samples collected at birth to 3 years.

To analyze the risk of later moderate to severe infections and systemic antibiotic prescriptions from early-life common infection burden from birth to 3 years, we separated early infections into 4 quartiles and into a high vs low group divided by a median split based on number of total infections. We analyzed the risk by using a continuous model per each early infection. We further analyzed each subtype of early infections relative to risk of moderate to severe infections and systemic antibiotic treatments. Viral airway pathogens between birth and 3 years were analyzed relative to later pneumonia risk after age 3 years as well. Estimates were derived from a quasi-Poisson regression model to deal with overdispersed data, ie, the variance is greater than the mean of the count distribution of the data. Using regular Poisson regression in this case would lead to false results with unrealistically small *P* values since the variance would be underestimated. We restricted the analysis to include children with full follow-up until at least the 10-year visit, and we performed a sensitivity analysis including children with more than 90% of diary data recorded between birth and 3 years. All analyses were conducted using R, version 4.0.3 (R Foundation). A 2-sided *P* < .05 was considered statistically significant.

## Results

Of the 700 included children, 642 had a clinical follow-up until at least age 10 years, and daily diary infection data from birth to 3 years were available for 614 (297 female [48.4%] and 317 male [51.6%]) of these 642 children; ie, 87.7% of the 700 children were included in the analysis (eFigure 1 in Supplement 1). As previously reported, there were no differences in baseline characteristics between the children having vs not having available diary data.^24^

We found a mean (SD) of 16.4 (8.0) infection episodes, and the most frequent type of infection was cold, with a mean (SD) of 12.3 (7.9) episodes per child from birth to 3 years. The distribution of diary-registered infection episodes have been previously published.^24^ After age 3 years until 10 or 13 years, the 614 children with follow-up experienced 268 moderate to severe infections (mean [SD], 0.44 [1.26] episodes per child), with pneumonia episodes constituting the largest proportion (208 of 268 [77.6%]). For systemic antibiotic episodes, we observed a mean (SD) of 2.32 (2.97) treatments per child from age 3 until 10 or 13 years.

There were no differences in social and environmental characteristics between children in the upper quartile (Q4) vs lower quartile (Q1) of total number of infections (mean [SD], 27.3 [4.6] vs 6.9 [2.8], respectively) between birth and 3 years except that more children in Q4 lived in an urban vs rural environment (99 of 153 [64.7%] in Q4 vs 73 of 154 [47.4%] in Q1; *P* = .004) (eTable 2 in Supplement 1). To mitigate the risk of confounding, all analyses were adjusted for sex, delivery mode, maternal smoking, Apgar score, child hospitalization, pregnancy interventions, maternal social circumstances, time to start of day care, older siblings, exposure to pets with fur, and living environment as described in detail in the Methods.

### Early Infection Burden vs Later Moderate to Severe Infections and Systemic Antibiotic Treatments

The total number of diary-registered infections from birth to 3 years was associated with a later increased risk of moderate to severe infection episodes from age 3 until 10 or 13 years (adjusted incidence rate ratio [AIRR], 1.05 ; 95% CI, 1.02-1.08; *P* < .001) and systemic antibiotic treatments (AIRR, 1.02; 95% CI, 1.01-1.04; *P* < .001) (Table 1). In a sensitivity analysis including all 700 children, we found similar associations between early vs later infection burden (AIRR, 1.05; 95% CI, 1.02-1.08; *P* < .001) and early infection burden and later risk of systemic antibiotic treatments (AIRR, 1.02; 95% CI, 1.01-1.04; *P* < .001).

**Table 1.** Number of Diary-Registered Infection Episodes From Birth to 3 Years and Later Risk of Moderate to Severe Infections and Systemic Antibiotic Treatments Between Age 3 and 10 or 13 Years

Infection types from birth to 3 y	Risk of moderate to severe infections from age 3 until 10 or 13 y		Risk of systemic antibiotic treatments from age 3 until 10 or 13 y	
	AIRR (95% CI)^a^	*P* value	AIRR (95% CI)^a^	*P* value
Total number of infection episodes	1.05 (1.02-1.08)	<.001	1.02 (1.01-1.04)	<.001
Subtype				
Cold	1.04 (1.01-1.07)	<.01	1.01 (1.00-1.03)	.047
Pneumonia	1.48 (1.34-1.62)	<.001	1.26 (1.17-1.35)	<.001
Gastric infection	1.03 (0.90-1.17)	.65	1.08 (1.01-1.45)	.02
Tonsillitis	1.01 (0.70-1.37)	.95	1.03 (0.87-1.19)	.75
Fever	1.08 (1.03-1.13)	.002	1.04 (1.01-1.07)	.002
Acute otitis media	1.08 (0.98-1.17)	.11	1.09 (1.05-1.14)	<.001

Abbreviation: AIRR, adjusted incidence rate ratio.

^a^ Estimates and 95% CIs are from adjusted quasi-Poisson regression models. Estimates were adjusted for fish oil and vitamin D interventions, number of older siblings, maternal social circumstances, time to start of day care, exposure to pets with fur during the first year, and living environment.

A high vs low infection burden before age 3 years divided into quartiles was associated with a later increased risk of moderate to severe infections from age 3 until 10 or 13 years (91 vs 45 episodes; Q4 vs Q1 AIRR, 3.02; 95% CI, 1.51-6.53; *P* = .003) and increased risk of later systemic antibiotic treatments (423 vs 324 episodes; Q4 vs Q1 AIRR, 1.59; 95% CI, 1.15-2.21; *P* = .006) (Table 2). In a sensitivity analysis including only children with more than 90% complete diary data from birth to 3 years, the results were similar, with a later increased risk of infection episodes (Q4 vs Q1 AIRR, 3.15; 95% CI, 1.43-8.04; *P* = .008) and risk of later systemic antibiotic treatment episodes (Q4 vs Q1 AIRR, 1.69; 95% CI, 1.16-2.50; *P* = .007). In another sensitivity analysis including only children from an urban living environment (n = 339), we found similar results between a high vs low infection burden from birth to 3 years and risk of moderate to severe infection episodes (Q4 vs Q1 AIRR, 2.66; 95% CI, 1.28-6.19; *P* = .01) and risk of systemic antibiotic treatment episodes (Q4 vs Q1 AIRR, 1.78; 95% CI, 1.22-2.67; *P* = .004).

**Table 2.** Daily Infection Load From Birth to 3 Years Defined as Quartiles vs Risk of Later Moderate to Severe Infections and Systemic Antibiotic Treatments Until Age 10 or 13 Years

Infection quartile for birth to 3 y	Mean (SD) diary-registered infections	Risk of moderate to severe infections from age 3 until 10 or 13 y		Risk of systemic antibiotic treatments from age 3 until 10 or 13 y	
		AIRR (95% CI)^a^	*P* value	AIRR (95% CI)^a^	*P* value
1 (lowest)	6.9 (2.8)	1 [Reference]	NA	1 [Reference]	NA
2	13.2 (1.5)	1.52 (0.69-3.49)	.31	1.16 (0.82-1.65)	.39
3	18.3 (1.8)	3.02 (1.53-6.48)	.003	1.32 (0.95-1.84)	.10
4 (highest)	27.3 (4.6)	3.02 (1.51-6.53)	.003	1.59 (1.15-2.21)	.006

Abbreviations: AIRR, adjusted incidence rate ratio; NA, not applicable.

^a^ Estimates and 95% CIs are from adjusted quasi-Poisson regression models. Estimates adjusted for fish oil and vitamin D interventions, number of older siblings, maternal social circumstances, time to start of day care, exposure to pets with fur during the first year, and living environment.

**Figure 1.**
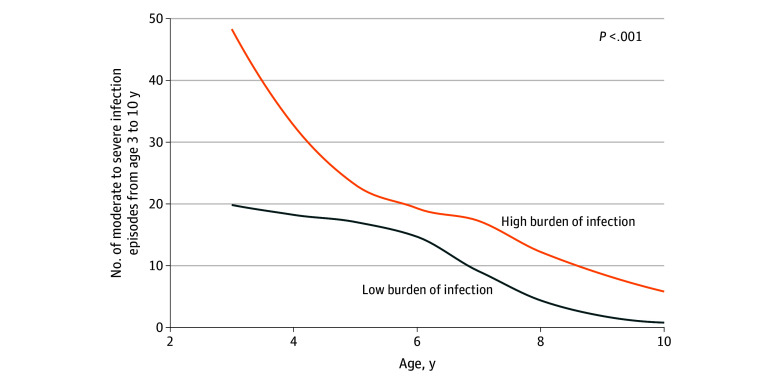
Number of Moderate to Severe Infection Episodes Per Year After Age 3 Years Illustrated for children with high (ie, median ≥16 episodes) vs low infection burden based on daily diary data in early childhood (birth to 3 years). The *P* value is from an adjusted quasi-Poisson regression model.

**Figure 2.**
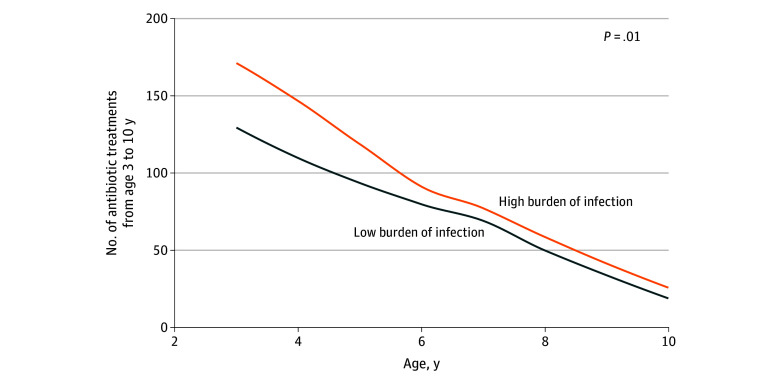
Number of Systemic Antibiotic Treatments Per Year After Age 3 Years Illustrated for children with high (ie, median ≥16 episodes) vs low infection burden based on daily diary data in early childhood (birth to 3 years). The *P* value is from an adjusted quasi-Poisson regression model.

When dividing the infection burden from birth to 3 years by a median split (≥16 episodes) in high-burden (n = 311) vs low-burden (n = 303) groups, we found that children with a high vs low burden had a later increased risk of moderate to severe infections (181 vs 87 episodes; AIRR, 2.39; 95% CI, 1.52-3.89; *P* < .001) (Figure 1) and increased risk of systemic antibiotic treatments (799 vs 623 episodes; AIRR, 1.34; 95% CI, 1.07-1.68; *P* = .01) (Figure 2). In a subanalysis, we investigated whether the association between early infections and later infections was only driven by airway infections and excluded all pneumonia episodes, leaving 60 infection episodes after age 3 years for the analysis. We found that each early infection episode from birth to 3 years was also associated with later infection risk without pneumonia between age 3 and 10 or 13 years (AIRR, 1.06; 1.02-1.10; *P* = .003) and that children with a high (≥16 episodes) vs low burden had an increased risk of later moderate to severe infections without pneumonia (AIRR, 2.17; 95% CI, 1.13-4.38; *P* = .02).

Subanalyses of the early infection types revealed that each cold, pneumonia, acute otitis media, fever, and gastric infection episode from birth to 3 years was associated with an increased risk of later systemic antibiotic treatment between age 3 and 10 or 13 years, whereas there was no association for tonsillitis (Table 1). In addition, early cold, pneumonia, and fever episodes from birth to 3 years were associated with an increased risk of later moderate to severe infections between age 3 and 10 or 13 years (Table 1).

### Early Airway Infection Episodes vs Later Pneumonia Risk

**Figure 3.**
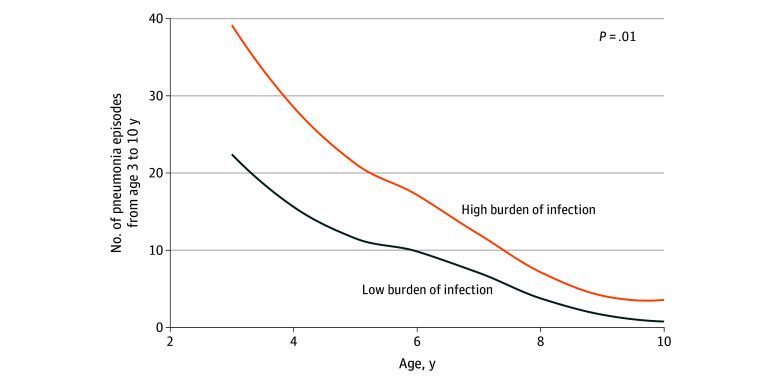
Number of Pneumonia Episodes Per Year After Age 3 Years Illustrated for children with high (ie, median ≥11 episodes) vs low burden of cold episodes based on daily diary data in early childhood (birth to 3 years). The *P* value is from an adjusted quasi-Poisson regression model.

The most prevalent type of infection registered in the diaries from birth to 3 years was cold, as previously illustrated for the COPSAC2010 cohort.^24^ The number of cold episodes before age 3 years was associated with an increased risk of later pneumonia until age 10 or 13 years (AIRR, 1.03; 95% CI, 1.00-1.07; *P* = .03). When grouping children by number of cold episodes into a high vs low group by the median (≥11 episodes), we found that children in the high group had an increased risk of later pneumonia episodes until age 10 or 13 years (AIRR, 1.99; 95% CI, 1.19-3.43; *P* = .01) (Figure 3).

In a similar analysis of early pneumonia burden, we investigated the number of diary-registered pneumonia episodes from birth to 3 years vs the risk of later pneumonia from age 3 to 10 or 13 years and found that each early pneumonia episode was associated with an increased pneumonia risk later in childhood (AIRR, 1.51; 95% CI, 1.35-1.67; *P* < .001). This finding was similar when further adjusting for asthma status at age 10 years (AIRR, 1.38; 95% CI, 1.24-1.52; *P* < .001). Due to a low number of episodes, instead of a median split, we divided the children into 2 groups on whether they had experienced any pneumonia episode (≥1 episode), which showed that the pneumonia (208 episodes) vs no pneumonia (406 episodes) group was associated with a higher later pneumonia risk (AIRR, 3.10; 95% CI, 1.92-5.08; *P* < .001). When dividing the children into 2 groups of having more than 1 pneumonia episode (83 children) from birth to 3 years vs having 1 or 0 episodes (531 children), the signal became even stronger with a higher effect estimate (AIRR, 4.06; 95% CI, 2.47-6.57; *P* < .001).

### Early Acute Airway Virus Episodes vs Later Pneumonia Risk

Among a subgroup of 309 children with troublesome lung symptoms who had an acute airway virus sample examined between birth and 3 years, we found that the number of specific airway viruses measured in the nasopharynx was associated with later pneumonia risk. This finding was significant for the burden of rhinoviruses (174 episodes; AIRR, 1.70; 95% CI, 1.34-2.12; *P* < .001) and enteroviruses (143 episodes; AIRR, 1.89; 95% CI, 1.37-2.58; *P* < .001) but not for RSV (117 episodes; AIRR, 1.14; 95% CI, 0.58-2.13; *P* = .69) (eFigure 2 in Supplement 1). Interestingly, the burden of enteroviruses was also associated with other types of moderate to severe infections when excluding pneumonia episodes (AIRR, 1.75; 95% CI, 1.04-2.82; *P* = .03), whereas rhinoviruses only showed a similar trend (AIRR, 1.52; 95% CI, 0.92-2.34; *P* = .08) but not RSV. All analyses were adjusted for the same covariates as above.

## Discussion

In this prospective child cohort study with more than 10 years of clinical follow-up, we found that common infections in early childhood were associated with a later risk of both moderate to severe infection diagnoses and systemic antibiotic treatments until age 10 or 13 years, independent of social and environmental risk factors. Interestingly, each infection episode in early childhood was associated with an increased later risk of moderate to severe infections and systemic antibiotic treatments, which was not only limited to children experiencing the highest infection burden in early life. We also found that cold, acute otitis media, pneumonia, gastric infection, and fever episodes in early childhood increased the later risk of either moderate to severe infections or systemic antibiotic treatments. Additionally, a high burden of early upper respiratory tract infection and LRTI episodes was associated with an increased risk of pneumonia later in childhood. Finally, a high burden of enterovirus or rhinovirus infection load, but not RSV, from acute airway samples in early childhood was associated with an increased risk of pneumonia episodes until age 10 or 13 years.

### Interpretation

To our knowledge, little is known about the association between the early burden of infections and later infection susceptibility from longitudinal child cohort studies. A previous study from 2008 of the German MAS-90 cohort^25^ tracked children with frequent common cold episodes from infancy (birth to 2 years of age) into preschool age (3-5 years) and school age (6-12 years). The investigators found that children from the upper tertile of common colds in infancy were also in the upper tertile at preschool and school age in 41.8% and 45.2% of the cases, respectively.^25^ These infection trajectories align with the findings from our study, although the MAS-90 study did not investigate the association with later LRTI risk or overall infection risk and antibiotic prescriptions. Interestingly, the MAS-90 investigators accounted for sociodemographic factors as well and could not identify specific risk factors that may explain the recurrent cold episodes seen among children from the upper tertile of colds in early life. These findings align with our study in which the burden of common early-life infections was associated with a later risk of infections independent of social and environmental risk factors, even when stratifying by living environment, which was the only significant risk factor between children in Q4 vs Q1 of early infections in our study.

The burden of early childhood infections seems to not only influence the risk of later moderate to severe infections and antibiotic treatments. In other studies, childhood infection burden has been directly associated with the development of atopic diseases, such as asthma and allergies,^4,5^ cardiometabolic risk factors,^6^ and mental disorders^7^ later in life. Interestingly, the adverse outcomes of a high infection burden seem to reach into adulthood as well. A longitudinal birth cohort, the Cardiovascular Risk in Young Finns Study, showed that the number of infection-related hospitalizations beginning at preschool age was associated with adverse metabolic parameters, such as body mass index and metabolic syndrome, in later adulthood.^6^ Furthermore, a nationwide Danish study using data from national registries showed that hospitalization for severe infections and less severe infections was associated with a subsequent development of any mental disorder and related medication prescription in adolescents.^7^ It is important for all pediatricians to discuss these disease trajectories associated with early-life infections with families,^26^ knowing that a high infection load in the first years of life may increase the risk of infections in later childhood and a range of other chronic disorders later in life. This knowledge may provide the basis for targeted and more focused disease prevention in specific children to address risk factors such as smoking and an unhealthy lifestyle. We also found that children born in an urban vs rural environment had a higher risk of developing infections as previously reported, which may be due to changes in diet composition.^27^

Most of the diary-registered infections from birth to 3 years and the later moderate to severe infections retrieved from *ICD-10* codes in our study were respiratory tract infections. We assessed common viral pathogens from airway samples collected at acute care visits, which showed that the number of early rhinovirus and enterovirus, but not RSV, episodes was associated with an increased risk of later pneumonia and that enterovirus episodes were associated with an increased risk of moderate to severe nonpneumonia infections without pneumonia episodes. All 3 are well-known viruses that cause acute respiratory illnesses in young children.^28,29^ However, in contrast to our study, another birth cohort following infants from birth to 2 years found that children with a first RSV LRTI were 3 times more likely to develop recurrent LRTIs compared with non-RSV LRTI,^29^ suggesting that this virus type is an important risk factor for later pneumonia as well. Our nonsignificant finding was probably due to an underpowered analysis of the subgroup of children (n = 309) with acute airway samples vs their study population (n = 1143),^29^ as RSV is known to often cause LRTI in young children. Finally, the associations between early infection burden and later moderate to severe infection risk and antibiotic treatment episodes were not only driven by respiratory tract infections. We also found an overall increased risk of later moderate to severe nonpneumonia infection and later antibiotic treatment episodes from the burden of early cold, acute otitis media, pneumonia, gastric infection, and fever episodes.

### Strengths and Limitations

The primary strength of this study is the thorough longitudinal clinical follow-up during at least 10 years from birth into later childhood, with detailed diagnosis information and medication prescriptions on every child included in the analysis. The children underwent several scheduled and emergency care visits in the COPSAC clinic, allowing for diagnostic consistency. Furthermore, we had unique day-to-day diary registrations of the most common infection types from birth to 3 years that thoroughly described early infection burden. The cohort is population based, so our findings should be generalizable. We accounted for several potential external risk factors, including social and environmental exposures, that were collected throughout the follow-up period for each child to mitigate the risk of confounding. Additionally, information on virus types from acute respiratory illness episodes during the first 3 years of life was collected, which allowed for a detailed investigation of specific airway pathogens.

The study also had some limitations. First, although we had a high follow-up rate of 92% until age 10 years, the study may be limited by attrition bias. Second, our cohort is limited to a Danish population; therefore, the findings may not be applicable to other populations worldwide. Finally, we only had a small subgroup of children fulfilling the troublesome lung symptom criterion for having an acute airway virus sample tested in early childhood.

## Conclusions

In this cohort study of 614 children with a 10- or 13-year follow-up and unique data collected from diaries kept in early childhood, we show that the burden of common infection episodes during the first years of life was associated with an increased risk of later moderate to severe infections and systemic antibiotic treatments independent of social and environmental risk factors. These findings may be important for prognosis and follow-up of children experiencing a high burden of common infections in early life.

## Notes

Supplement 1. **eTable 1.** Infection Diagnoses Including *ICD-10* Codes From Age 3 to 10 or 13 Years **eTable 2.** Baseline Characteristics of the Q1 vs Q4 Infection Groups Based on Diary Data From Birth to 3 Years **eFigure 1.** Flowchart of the Study Participants **eFigure 2.** Risk of Pneumonia Episodes After Age 3 Years by Specific Acute Airway Infection Virus Episodes From Birth to 3 Years

Supplement 2.
Data Sharing Statement
