# Supplementary material for: Burden of Infections in Early Life and Risk of Infections and Systemic Antibiotics Use in Childhood
Source: JAMA Netw Open. 2025 Jan 6;8(1):e2453284. doi: 10.1001/jamanetworkopen.2024.53284 (PMC11704971; doi:10.1001/jamanetworkopen.2024.53284)
Supplement: Supplement 1. — eTable 1. Infection Diagnoses Including ICD-10 Codes From Age 3 to 10 or 13 Years eTable 2. Baseline Characteristics of the Q1 vs Q4 Infection Groups Based on Diary Data From Birth to 3 Years eFigure 1. Flowchart of the Study Participants eFigure 2. Risk of Pneumonia Episodes After Age 3 Years by Specific Acute Airway Infection Virus Episodes From Birth to 3 Years [file jamanetwopen-e2453284-s001.pdf]

Supplemental Online Content

Brustad N, Buchvald F, Jensen SK. Burden of infections in early life and risk of infections and systemic antibiotics use in childhood. *JAMA Netw Open*. 2025;8(1):e2453284.
doi:10.1001/jamanetworkopen.2024.53284

eTable 1. Infection Diagnoses Including *ICD-10* Codes From Age 3 to 10 or 13 Years

eTable 2. Baseline Characteristics of the Q1 vs Q4 Infection Groups Based on Diary Data From Birth to 3 Years

eFigure 1. Flowchart of the Study Participants

eFigure 2. Risk of Pneumonia Episodes After Age 3 Years by Specific Acute Airway Infection Virus Episodes From Birth to 3 Years

This supplemental material has been provided by the authors to give readers additional information about their work.

© 2025 Brustad N et al. *JAMA Network Open.*

eTable 1. Infection Diagnoses Including *ICD-10* Codes From Age 3 to 10 or 13 Years

Diagnoses	ICD-10 codes
Sepsis	A40.xxxx, A41.xxxx, R65.10xx, R65.11xx, R65.20xx, R65.21xx, T81.12XA, T81.12XD, T81.12XS
Pneumonia	J12.xxxx -J18.xxxx
Acute gastroenteritis	A01.x-A09.x, R11.1, R11.2
Pyelonephritis	N11.0, N11.8, N11.9, N10, N15.1, N28.84, N28.85, N28.86, N12, N16, N15.9, N13.6
Meningitis or encephalitis	A02.21, A39.0, A83-A87, A88.8, A89, B02.1, B00.3, B26.1, A51.41, A52.13, A54.81, A27.81, G00-G05, A39.81, B00.4, B06.01, B10.01, B10.09, A92.31, B26.2, A52.14, B58.2, G92
Cellulitis, soft tissue infection or herpes zoster	A46, A48.0, K94.02, K94.12, L03, L02, K12.2, M72.6, I96, B02.x
Septic arthritis or osteomyelitis	A02.23, B06.82, A54.4, A02.24, H05.029, M27.2, M00, M01, M46.2, M86

© 2025 Brustad N et al. *JAMA Network Open.*

Endocarditis	A39.51, B33.21, A52.03, A54.83, I33.0, I39, I33.9, I40.0

© 2025 Brustad N et al. *JAMA Network Open.*

eTable 2. Baseline Characteristics of the Q1 vs Q4 Infection Groups Based on Diary Data From Birth to 3 Years

	Infection groups age 0-3 years		
Characteristics	Q1 (n=154)	Q4 (n=153)	P value
Delivery mode: Section, n	40	32	0.30
Sex: Males, n	80	79	0.96
Apgar score at 1 min, mean (SD)	9.48 (1.09)	9.42 (1.32)	0.64
Maternal social circumstances, mean (SD)	-0.1	0.04	0.21
Pregnancy vitamin D intervention, n	63	56	0.73
Pregnancy fish oil intervention, n	82	71	0.21
Urban living environment at birth, n	73	99	0.004
Time to daycare start, mean (SD)	0.89 (0.21)	0.9 (0.20)	0.50

© 2025 Brustad N et al. *JAMA Network Open.*

Number of older siblings, mean (SD)	0.80	0.82	0.81
Exposure to furred pets during 1st year, n	122	126	0.73
Hospitalized at birth, n	21	21	0.98
Maternal smoking during pregnancy, n	15	11	0.42

© 2025 Brustad N et al. *JAMA Network Open.*

eFigure 1. Flowchart of the Study Participants. The low and high groups are defined by a burden of infection at age 0-3 years above vs. below the median of 16 diary-registered episodes.

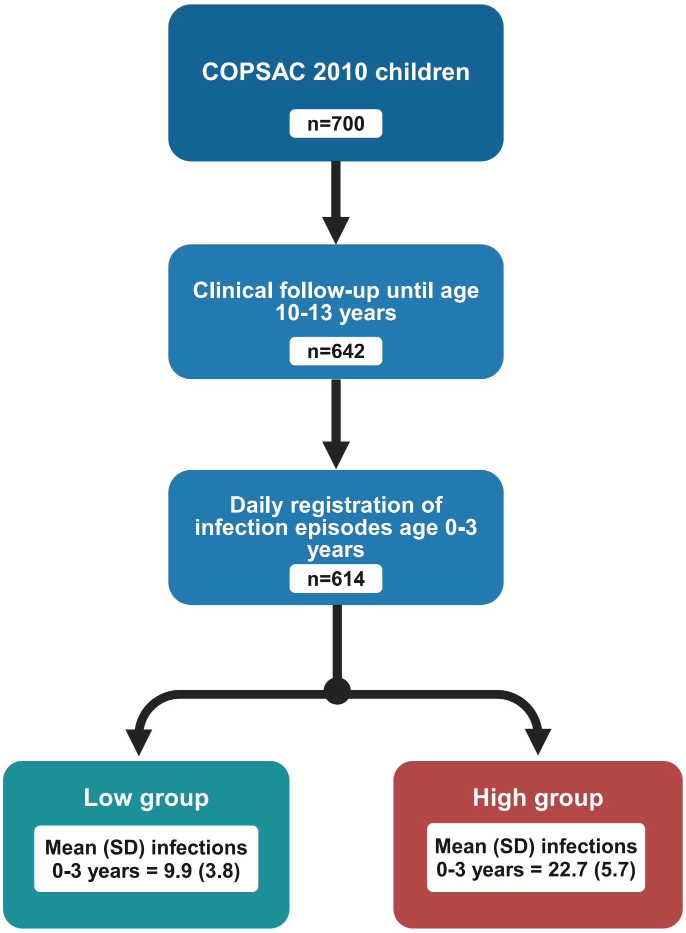

© 2025 Brustad N et al. *JAMA Network Open.*

eFigure 2. Risk of Pneumonia Episodes After Age 3 Years by Specific Acute Airway Infection Virus Episodes From Birth to 3 Years. Estimates and 95% CIs from an adjusted Quasi-Poisson regression model.

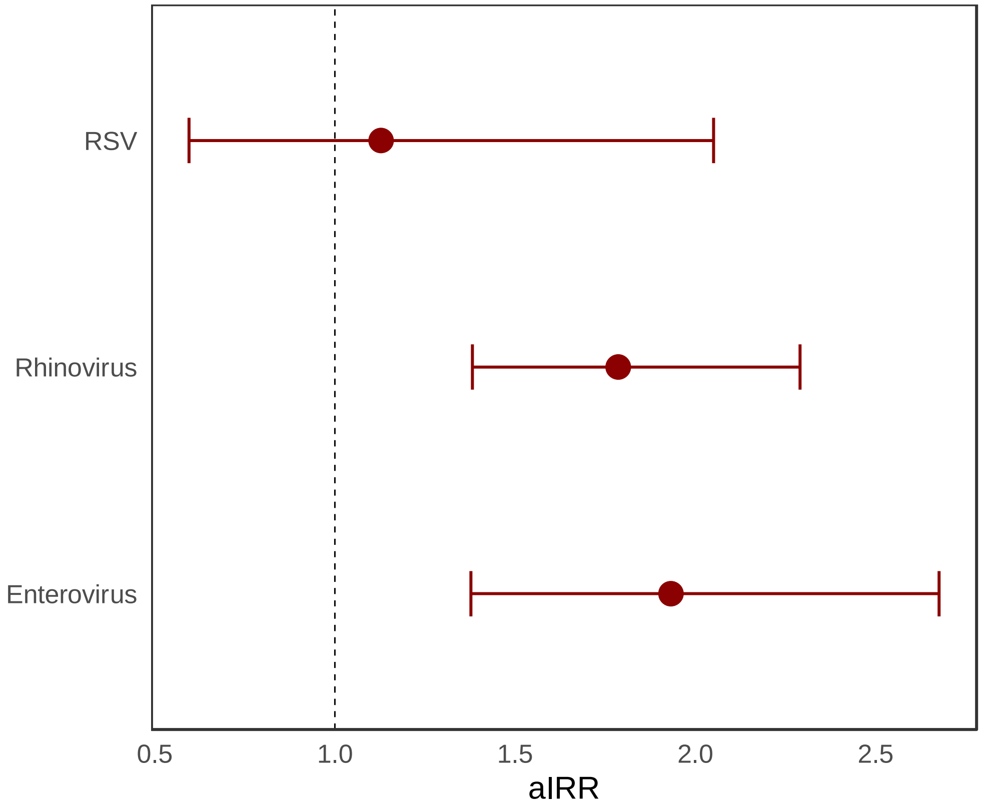

© 2025 Brustad N et al. *JAMA Network Open.*
