# Supplementary material for: Burden of Infections in Early Life and Risk of Infections and Systemic Antibiotics Use in Childhood
Source: JAMA Netw Open. 2025 Jan 6;8(1):e2453284. doi: 10.1001/jamanetworkopen.2024.53284 (PMC11704971; doi:10.1001/jamanetworkopen.2024.53284)
Supplement: Supplement 2. — Data Sharing Statement [file jamanetwopen-e2453284-s002.pdf]

Data Sharing Statement

Brustad. Burden of Infections in Early Life and Risk of Infections and Systemic Antibiotics Use in Childhood. *JAMA Netw Open*. Published January 06, 2025.
doi:10.1001/jamanetworkopen.2024.53284

Data
Data available: Yes
Data types: Deidentified participant data
How to access data: By request to nicklas.brustad@dbac.dk
When available: With publication

Supporting Documents
Document types: None

Additional Information
Who can access the data: researchers whose proposed use of the data has been approved
Types of analyses: for a specified purpose
Mechanisms of data availability: with a signed data access agreement
